# Integrative analysis of single-cell and bulk RNA sequencing unveils the senescence landscape in ischemic stroke

**DOI:** 10.18632/aging.204804

**Published:** 2023-06-28

**Authors:** Longhui Fu, Beibei Yu, Yongfeng Zhang, Shuai Cao, Boqiang Lv, Yunze Tian, Huangtao Chen, Shijie Yang, Yutian Hu, Jinghua Hua, Pengyu Ren, Jianzhong Li, Shouping Gong

**Affiliations:** 1 Xi’an Jiaotong University, Xi’an, China; 2 Department of Neurosurgery, Second Affiliated Hospital of Xi’an Jiao Tong University, Xi’an, China; 3 Department of Thoracic Surgery, Second Affiliated Hospital of Xi’an Jiao Tong University, Xi’an, China; 4 Department of Orthopedics, Civil Aviation General Hospital, Chaoyang, Beijing, China; 5 Xi’an Medical University, Xi’an, China

**Keywords:** ischemic stroke, aging, cellular senescence, single-cell RNA-seq, bioinformatics

## Abstract

Ischemic stroke (IS) is a fatal neurological disease that occurs when the blood flow to the brain is disrupted, leading to brain tissue damage and functional impairment. Cellular senescence, a vital characteristic of aging, is associated with a poor prognosis for IS. This study explores the potential role of cellular senescence in the pathological process following IS by analyzing transcriptome data from multiple datasets (GSE163654, GSE16561, GSE119121, and GSE174574). By using bioinformatics methods, we identified hub-senescence-related genes such as *ANGPTL4, CCL3, CCL7, CXCL16,* and *TNF* and verified them using quantitative reverse transcription polymerase chain reaction. Further analysis of single-cell RNA sequencing data suggests that MG4 microglial is highly correlated with cellular senescence in MCAO, and might play a crucial role in the pathological process after IS. Additionally, we identified retinoic acid as a potential drug for improving the prognosis of IS. This comprehensive investigation of cellular senescence in various brain tissues and peripheral blood cell types provides valuable insights into the underlying mechanisms of the pathology of IS and identifies potential therapeutic targets for improving patient outcomes.

## INTRODUCTION

Stroke is among the most fatal neurological diseases, and it is the second leading cause of death in those aged >60 years and the fifth leading cause of death in those aged <15 years [1–3]. Strokes are clinically classified into ischemic stroke (IS), hemorrhagic stroke, and transient ischemic attack, with IS accounting for 80% of all stroke cases [3, 4]. IS is not only a major cause of death but is also responsible for a significant number of disability-adjusted life years, which increased by 138.6% from 1990 to 2019 [5]. Therefore, improving the prognosis of IS is crucial for alleviating the disease burden.

Senescence is a fundamental biological process characterized by a general decline in tissue function, increased susceptibility to neurological diseases, and decreased resistance to inflammation and infection [6]. Typically, IS is accompanied by accelerated sensory-motor and neurocognitive decline, which are signs of senescence [7, 8]. Accordingly, advanced age is a known risk factor for IS [9]. Furthermore, IS is strongly associated with cellular senescence, a major cause of aging [10]. Cellular senescence refers to the permanent state of cell cycle arrest, which is a defense mechanism that prevents unwanted damage to cells [11]. The inability of cells to re-enter the cell cycle in response to irreversible growth arrest, resistance to apoptosis, production of the senescence-associated secretory phenotype (SASP), mitochondrial dysfunction, and changes in DNA and chromatin levels are common pathophysiological processes of cellular senescence [12]. High levels of inflammatory cytokines and SASP have been detected in the IS-pedunculated region [13]. Various studies have shown that cellular senescence intervention improves the prognosis of patients with IS and is a promising therapeutic approach [14, 15]. There are good reasons to believe that cellular senescence plays an important role in the pathophysiological process of IS, and there are solid grounds for the assertion that cellular senescence is crucial to the pathophysiology of IS.

**Figure 1:**
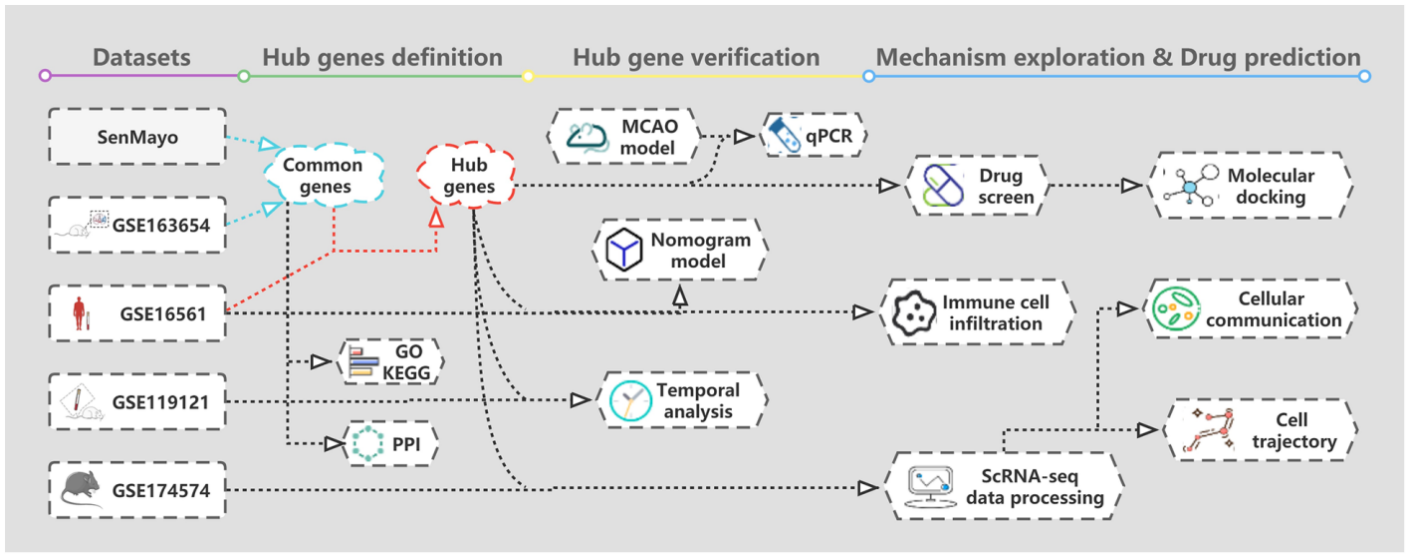
The flowchart of data preparation and analysis.

Identifying senescent cells *in vivo* remains challenging, although cellular senescence can drive a variety of age-related disease manifestations through aging-related secretory phenotypes. Recently published gene sets related to senescent cells can aid in identifying *in vivo* cellular senescence [16]. Moreover, senescence can vary significantly in different cell types. The senescence of endothelial, smooth muscle and immune cells is believed to participate in the senescence of blood vessels, and the senescence of immune cells is believed to promote the aging of other cell types [17, 18]. Additionally, the senescence of neurons and glial cells is widespread in neurodegenerative diseases [19]. However, few studies have examined cellular senescence after IS, and there is a lack of research on the relationship between cellular senescence and a wide range of cell types in the brain. In this study, we identified hub genes for cellular senescence in IS using bioinformatics and experimental validation and explored their biological pathways. Using single-cell RNA sequencing (scRNA-seq), we evaluated the hub senescence-related gene (HSRG) expressions in various cell types and mapped the developmental trajectories of microglia and cellular communication networks. Finally, we predicted potential therapeutic drugs based on the HSRGs. The approach used in this study is depicted in the flow diagram (Figure 1).

## MATERIALS AND METHODS

### Microarray datasets

SenMayo is a recently published gene set that includes 125 and 118 unrepeatable genes in humans and mice, respectively [16]. The gene set was downloaded from the supplementary information of the original article. The Gene Expression Omnibus (“ http://www.ncbi.nlm.nih.gov/geo/”) is an open-source database that provides gene expression profiles for our study. Four datasets including GSE163654, GSE16561, GSE119121, and GSE174574 were used (Table 1). Bulk RNA-sequencing (bulk RNA-seq) of brain tissue from three sham-operated rats and three middle cerebral artery occlusion (MCAO) rats in GSE163654 was used for differential expression analysis. GSE16561 contains bulk RNA-seq data of peripheral blood from 39 patients with IS and 24 patients with normal groups, which were used for expression and immune cell infiltration analyses. GSE119121 contains bulk RNA-seq data from rat peripheral blood from the MCAO and sham groups used for the temporal analysis of gene expression. Finally, scRNA-seq data of the brain tissue from GSE174574 with three sham group mice and three MCAO group mice were processed and used for cell communication analysis.

**Table 1:** Detailed information of the gene expression matrixes and platform.

**GEO dataset**	**Platform**	**Species**	**Tissue**	**Country**	**Author**
GSE163654	GPL17117	Rat	Penumbras tissue of brains	Canada	Tymianski M et al.
GSE16561	GPL6883	Human	Peripheral blood	USA	Barr TL et al.
GSE119121	GPL6247	Rat	Blood	Belgium	Dagonnier M et al.
GSE174574	GPL21103	C57BL/6	Brain	China	Zheng K, Hao J

### Differential expression analysis

The R software (v4.2.1, R Foundation, Vienna, Austria) was used for all analyses and visualizations in this study. To create the analysis matrix, all original bulk RNA-seq matrices were normalized and coupled with the associated RNA probes. Data with non-mRNA expression loss and no corresponding gene names were excluded. The differentially expressed genes (DEGs) were screened using the criteria |log2 (fold change) | >0.5 and a p-value of <0.05. Heatmaps and volcano plots were generated using the “heatmap” and “ggplot2” packages, respectively. Finally, a Venn diagram was created using the website http://www.bioinformatics.com.cn/ for the Venn analysis.

### Pathway enrichment analysis and protein-protein interaction network

Gene ontology (GO) and Kyoto Encyclopedia of Genes and Genomes (KEGG) pathway analysis of DEGs were performed and visualized using the “org.Mm.eg.db” and “clusterProfiler” packages. The free website, STRING (https://www.string-db.org/), was used to analyze functional protein association networks of DEGs. A minimum interaction score of ≥0.150 was defined as the cut-off value, and the resulting protein-protein interaction network was visualized using Cytoscape software. Finally, the hub genes were ascertained by visualizing the bulk RNA-seq of DEGs in GSE16561 using the “reshape2” and “ggpubr” packages.

### Animal and establishment of the MCAO model

The Medical Experimental Animal Center (Xi’an Jiaotong University) provided 12 pathogen-free male Sprague-Dawley rats (weight: 280–300 g). A modified Zea-Longa model, in which the coil occlusion was permanently placed in the middle cerebral artery, was used to create a rat permanent MCAO model [20]. The rats (n = 8) were randomly allocated to either the sham group or the MCAO group, with four rats in each group. The Longa scale was used to assess the neurobehavioral scores of rats in each group two hours after MCAO. Animals with no neurological impairment following surgery were excluded from the study. The rats were euthanized three days after the operation via intraperitoneal injection. The brains were removed and sliced before being put in 2% triphenyl tetrazolium chloride (TTC) (Solarbio Life Science, Beijing, China) and incubated at 37° C for 30 minutes.

### Quantitative reverse transcription polymerase chain reaction (RT-qPCR)

Three rats from each group were anesthetized 48 hours after surgery, and tissue samples were collected from the ischemic penumbra. The samples were immediately stored in liquid nitrogen, and total RNA was extracted from each sample using the TRIzol reagent (Sinopharm Chemical Reagents Co., Ltd., China). The extracted RNA was reverse-transcribed into complementary DNA using SweScript All-in-One RT SuperMix (Wuhan Saiwei Biotechnology Co., Ltd., China). Table 2 shows the primer sequences used in the study. The 2-Ct method was used to calculate the relative mRNA expression, which was then compared to that of the normal group (glyceraldehyde 3-phosphate dehydrogenase mRNA expression). A student’s t-test was used for statistical comparisons, and differences with a p-value of <0.05 were considered statistically significant.

**Table 2:** Specific primers used for quantitative real-time PCR.

**Gene**	**Forward**	**Reverse**
*Angptl4*	CATGGCTGCCTGCGGTAACG	AGTTGCTGGATCTTGCTGTTCTGAG
*Ccl3*	CACCGCTGCCCTTGCTGTTC	GGAATTTGCCGTCCATAGGAGAAGC
*Ccl7*	GATCTCTGCCGCGCTTCTGTG	TGGATGAATTGGTCCCATCTGGTTG
*Cxcl16*	CAGTTTCAGAGCACCCAGCAGTC	GCCTAGCCTCCAGACCATAGCC
*Tnf*	CACCACGCTCTTCTGTCTACTGAAC	TGGGCTACGGGCTTGTCACTC

### Construction of a prediction model

The nomogram model, calibration, decision, and clinical impact curves were based on the expression data of HSRGs in GSE16561, implemented by the “rms” and “rmda” packages. The receiver operating characteristic (ROC) curve was also plotted through the “ROCR” package, and the calibration, decision curve analysis (DCA), and clinical impact curves were drawn.

### Temporal analysis of expression

GSE119121 contains the bulk RNA-seq of MCAO rats at different time points, and the DEGs expression was described by a heatmap and a violin plot using the “heatmap,” “reshape2,” and “ggpubr” packages. Simultaneously, the mean value of hub gene expression at different time points was calculated, and a line graph was drawn.

### Immune cell infiltration analysis

To analyze immune cell infiltrations in GSE16561 and calculate merged expression data, we used the CIBERSORT method, which is a technique for analyzing different immune cell types in tissues [21]. The samples were filtered using a p-value of <0.05, and a bar plot was generated to show the percentage of each immune cell type in each sample. The “pheatmap” package was used to generate a heatmap of the 22 immune cells and a heatmap describing the hub gene expression in immune cells as well. The package “vioplot” was used to compare and visualize the levels of 22 immune cells in IS and normal samples. Using the “corrplot” package, a correlation heatmap was generated that revealed the correlation of 22 different types of infiltrating immune cells.

### ScRNA-seq data processing and cell communication analysis

GSE174574 contains the scRNA-seq of three sham group mice and three MCAO group mice. ScRNA-seq data were processed using the “Seurat” package for unsupervised graph-based clustering before analysis [22]. The following were the screening criteria for the cells examined: Cells with 500–6,000 unique molecular identifiers and 35% of mitochondrial genes judged to be of high quality were eliminated from further research. The normalized data function was used to normalize the quality-controlled data, and then the find variable features tool was used to select 2000 highly variable genes. The mutual principal component analysis tool “Seurat” was used to integrate the data. The proportion of cells was determined by selecting the top 20 main components for the visualization of dimensionality reduction using uniform manifold approximation and projection (UMAP). The “SingleR” package was used for cell type identification, in which “MouseRNAseqData” was used as a reference. Additionally, the “cellcall” package was used to infer intercellular communication [23]. To determine differentiation trajectories for major clusters with large cell numbers, the “monocle3” package was used for cell trajectory analysis [24].

### Drug screening and molecular docking

The DSigDB database contains the Food and Drug Administration-approved drugs and experimental compounds (http://tanlab.ucdenver.edu/DSigDB) and is a free website with the DSigDB interface (https://maayanlab.cloud/Enrichr/). Drugs and compounds were predicted using Enrichr, based on hub genes. The screening criterion was adj. p <0.05, and the ranking was based on the comprehensive score. The protein was converted to the PDBQT file format so that AutoDock 4 software could recognize and read the modified protein. To prepare the ligands for docking, charges were added and optimized. Three PDBQT files were identified: rigid DEG proteins, flexible proteins, and drug ligands. Finally, we used AutoDock 4 software to perform molecular docking.

## RESULTS

### Identification of the senescence-related genes (SRGs)

**Figure 2:**
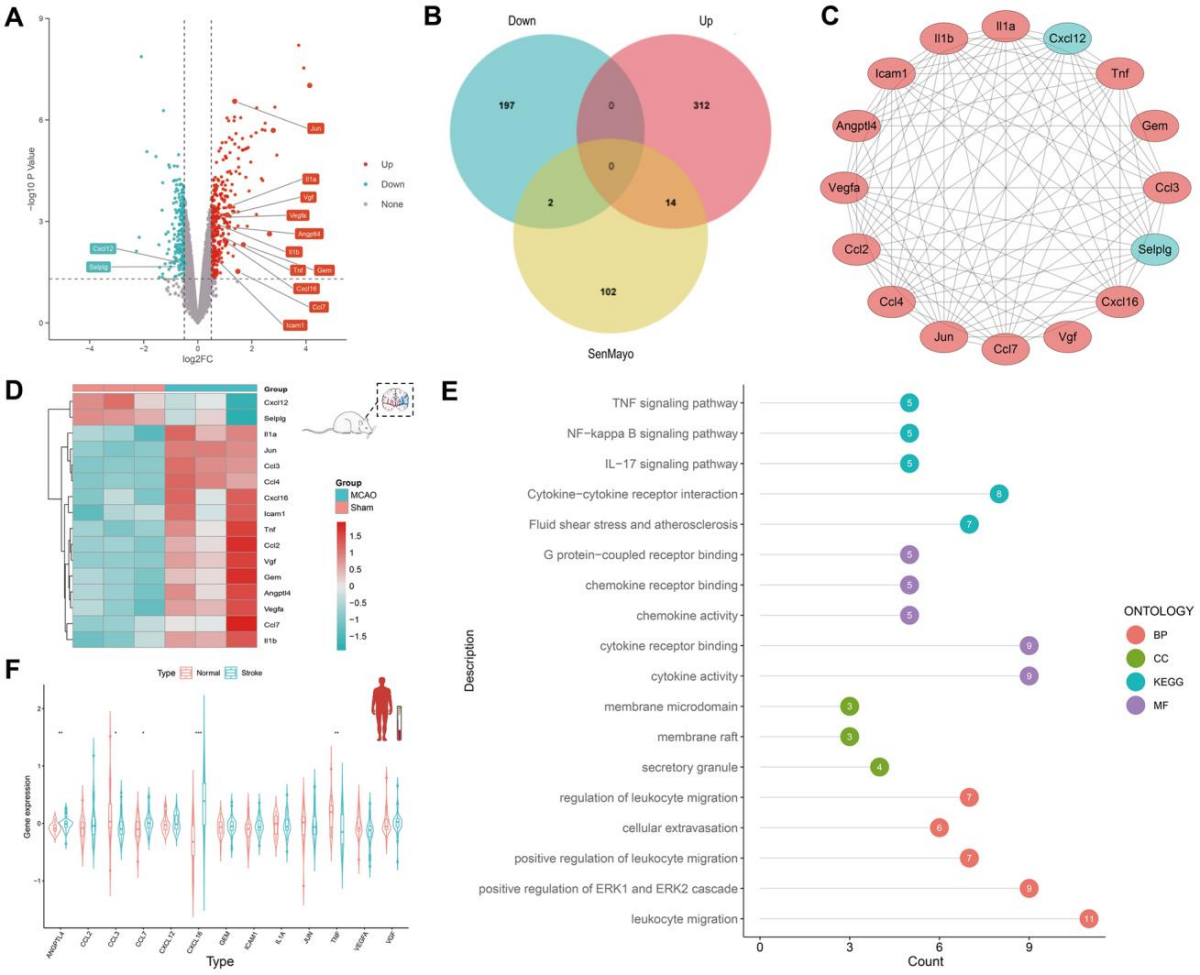
**Discovery of SRGs in rat MCAO model and human peripheral blood.** (**A**) The volcano plot for DEGs of brain tissue in GSE163654. The genes related to cellular senescence were labeled. Red represents high gene expression and blue represents low expression. (**B**) The Venn plot for the distribution of DEGs. (**C**) The protein-protein interaction network for SRGs. (**D**) The heatmap for SRGs in GSE163654. (**E**) GO/KEGG pathway analysis and protein interaction network of SRGs. The color of the proteins corresponds to the pathway and the number shows the count of genes. (**F**) The violin plot for SRGs of human peripheral blood in GSE16561. *p < 0.05, **p < 0.01, ***p < 0.001.

The DEGs between the six-hour rat MCAO groups and sham groups in GSE163654 were discovered and are shown in a volcano plot (Figure 2A). Among them, 326 genes were upregulated and 199 genes were downregulated. Subsequently, according to the Venn plot, 14 upregulated DEGs (*CCL3, Jun, CCL4, Il1a, VGF, VEGFA, IL1B*, *ANGPTL4*, *TNF*, *CCL2*, *CXCL16*, *GEM, ICAM1*, *CCL7*) and two downregulated SRGs (*CXCL12, SELPLG*) were involved in the senescence-related SenMayo dataset (Figure 2B). Specifically, 92 edges were linked between 16 corresponding proteins in the protein-protein interaction network (Figure 2C). A heatmap shows the expression of these 16 genes (Figure 2D). Based on these 16 genes, GO/KEGG functional enrichment analysis was performed. The subsequent GO/KEGG functional enrichment analysis showed that these genes were highly enriched in leukocyte migration, positive regulation of the ERK1 and ERK2 cascades, cytokine activity, and cytokine receptor binding (Figure 2E). Finally, to identify the conservation of these genes between species, we compared the expression of these 16 genes in human peripheral blood between the IS and normal groups (Figure 2F). Thirteen of these 16 genes were expressed in both mice and humans, and five (*ANGPTL4*, *CCL3*, *CCL7*, *CXCL16,* and *TNF*) showed statistically significant differences.

### SRGs expression in the peripheral blood of the rat

**Figure 3:**
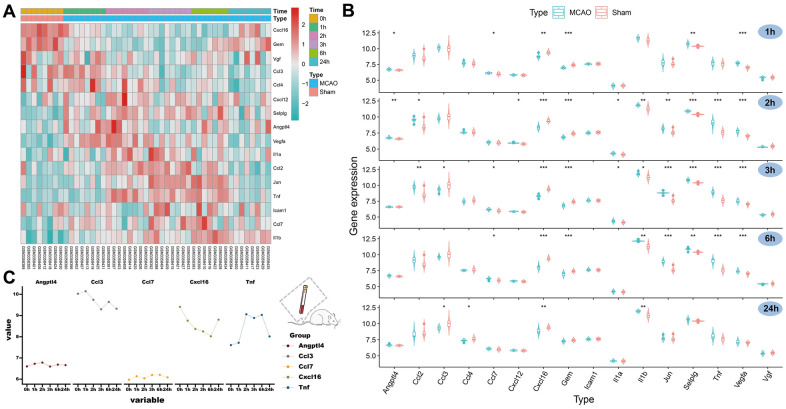
**Expression of SRGs in the rat peripheral blood and identification of HSRGs.** (**A**) The heatmap for SRGs in GSE119121 at different time points. (**B**) The violin plot for SRGs in GSE119121. (**C**) The line graph describes the variation trend of HSRGs expression at different time points.

By analyzing the GSE119121 expression matrix, we visualized the expression of these 16 SRGs in the peripheral blood of the MCAO and sham groups (Figure 3A). We further compared the expression trends of these SRGs at five different time points (1, 2, 3, 6, and 24 hours) (Figure 3B). Notably, *CXCL16* expression and *GEM* decreased in the MCAO group but tended to recover after 24 hours. The expression levels of *VGF*, *CCL3,* and *CCL4* decreased at later time points. Meanwhile, the expression levels of the other 11 genes increased at different time points within 24 hours in the MCAO group, and most of them recovered 24 hours after the operation. Additionally, the expression of five SRGs was significantly different at some points after the MACO operation compared with that before the operation. Finally, a line graph was drawn to describe the variation trends of the five hub genes (Figure 3C). Thus, *ANGPTL4*, *CCL3*, *CCL7*, *CXCL16,* and *TNF* have better species conservation in the peripheral blood of rats and humans and were identified as HSRGs for further research.

### Validation of HSRGs by RT-qPCR in the MCAO model

**Figure 4:**
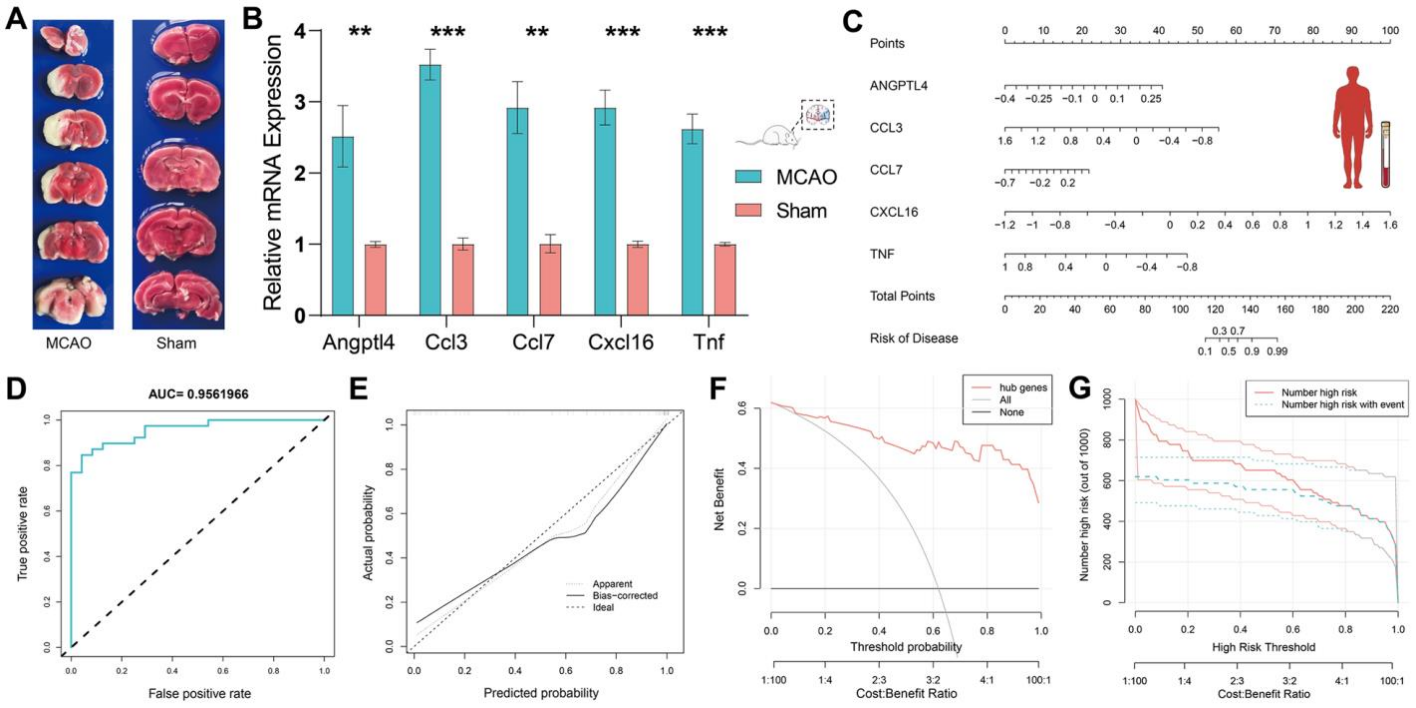
**Validation of HSRGs by rat MCAO model and construction of prediction model.** (**A**) TTC staining verification of rat MCAO model. (**B**) Validation of quantitative real-time PCR analysis. (**C**) Nomogram of HSRGs for predicting IS. A calibration curve (**D**), Clinical decision analysis (**E**, **F**), and ROC curve (**G**) of the nomogram.

We performed RT-qPCR in the MCAO model to demonstrate the critical role of HSRGs in IS. To verify the success of our MCAO model, TTC staining was performed on the brain tissue of MCAO rats (Figure 4A). TTC staining stains the normal brain tissue red, while the infarct lesions appear white, allowing a good evaluation of the infarct in the brain. According to the RT-qPCR results, the expression levels of all five genes differed significantly (p <0.05) (Figure 4B). Gene expression levels were higher in the brain tissues of rats in the MCAO model than in the sham group, and the expression trend was consistent with that shown in Figure 2D between the MCAO and sham groups in GSE163654.

### Construction of a clinical prediction model

Based on the expression levels of HSRGs in GSE16561, we constructed a nomogram prediction model (Figure 4C). To verify the effectiveness of the model, ROC, calibration, DCA, and clinical impact curves were plotted (Figure 4D–4G). The area under the curve (AUC) of the prediction model was approximately 0.956, and the calibration curve showed good calibration. The DCA curve showed that this predictive model could yield significantly greater net benefits for making clinical decisions. In terms of the clinical impact curve, the prediction model determined that the population at risk for IS was strongly matched to the actual population when the threshold probability was >65% of the predicted score probability value, confirming the good clinical efficiency of the prediction model.

### Immune cell infiltration analysis

**Figure 5:**
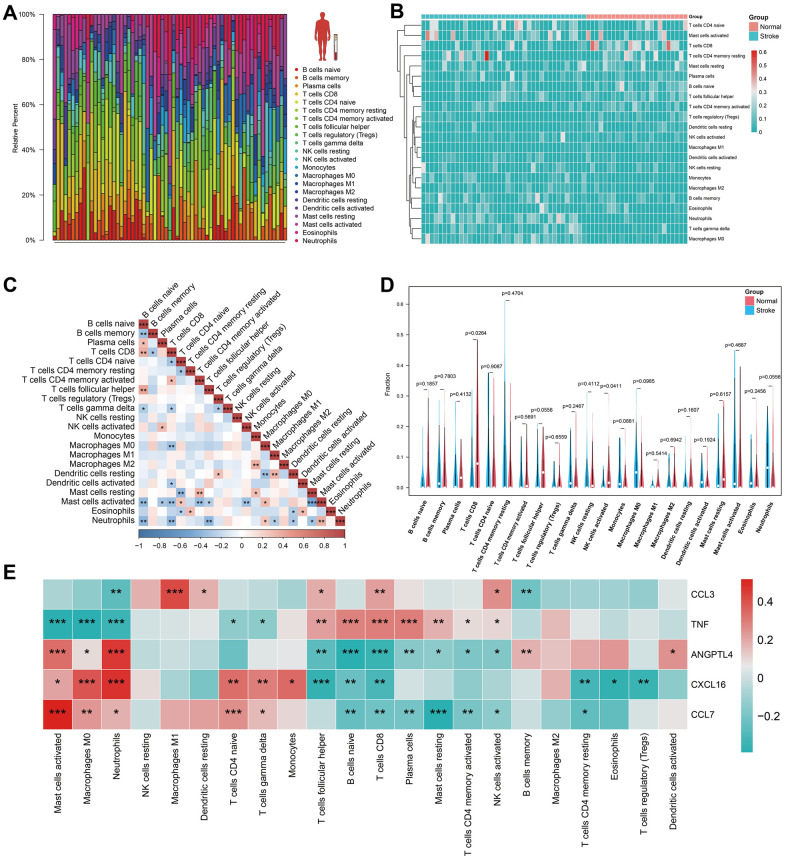
**Immune cell infiltration analysis in human peripheral blood.** (**A**, **B**) The landscape of immune infiltration between IS and normal groups in GSE16561. (**C**) Correlation matrix of all 22 immune cell subtype compositions. Higher, lower, and the same correlation levels are displayed in red, blue, and white. (**D**) Comparison of 22 immune cell subtypes between patients in IS and normal groups. (**E**) The heatmap for HSRGs in 22 immune cell subtype compositions.

The CIBERSORT algorithm was used to predict immune cell infiltration in the IS and normal groups. The bar plot and heatmap displayed the percentage of each of the 22 types of immune cells in each human blood sample from GSE16561 (Figure 5A, 5B). Correlation analysis of immune cells revealed that resting mast cells and activated mast cells had the most significant negative correlation, while naïve B cells and CD8 T cells, follicular helper cells, resting mast cells and activated CD4+ memory T cells, M2 macrophages and monocytes, resting dendritic cells and M1 macrophages, neutrophils and activated mast cells had a significant positive correlation (Figure 5C). The violin plot of the immune cell infiltration difference showed that, in comparison to the normal group, patients with IS had lower levels of CD8+ T cells and activated NK cells (Figure 5D). Finally, we analyzed HSRG expression in 22 types of immune cells (Figure 5E).

### ScRNA-seq reveals the cellular senescence pattern after IS

**Figure 6:**
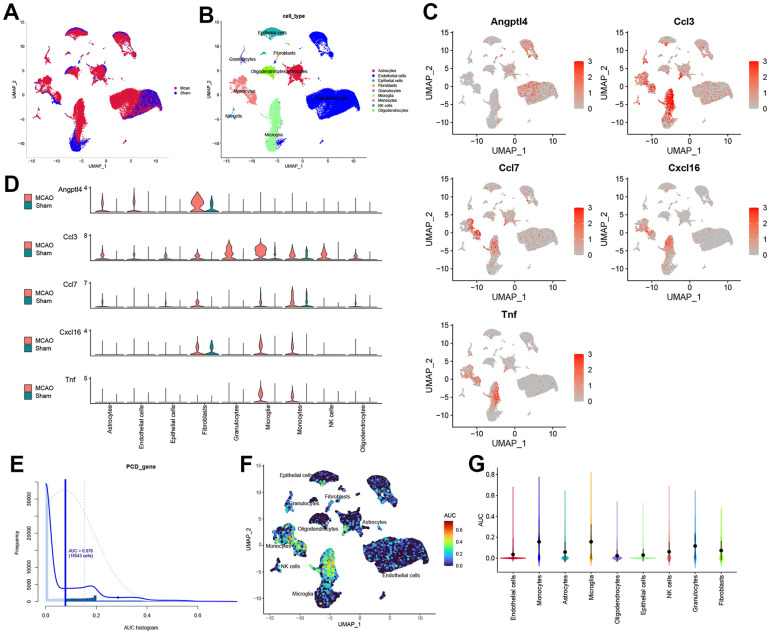
**The scRNA-seq reveals the expression of HSRGs in mouse brains.** (**A**) Cluster analysis of scRNA-seq in GSE174574 dataset. Red represents the cells in the MCAO group and blue represents the cells in the Sham group. (**B**) Cell cluster identification was obtained in (**A**). Different colors represent different cell clusters, with a total of 9 identified. (**C**) Distribution of HSRGs expression in different cell clusters. Compared with the Sham group, red represents the high expression of genes in the IS group. (**D**) Quantified expression of HSRGs in different cell clusters. (**E**) The distribution of cell AUC value, an AUC value greater than 0.078 were adopted. (**F**) Distribution of HSRGs expression in different cell clusters based on AUC value. (**G**) Quantified AUC value of HSRGs in different cell clusters.

Cell clusters were identified by UMAP analysis in MCAO and sham-operated mice (Figure 6A). We further annotated the cell clusters through the “SingleR” package and mapped them to the UMAP (Figure 6B). Nine cell clusters were identified: astrocytes, endothelial cells, epithelial cells, fibroblasts, granulocytes, microglia, monocytes, natural killer cells, and oligodendrocytes. Subsequently, HSRG expression in each cell cluster was mapped onto UMAP diagrams and quantified (Figure 6C, 6D). Finally, we used the “AUCell” package to evaluate the total hub gene expressions in all types of cells and cells with an AUC value greater than 0.078 were adopted (Figure 6E). Significantly, HSRGs were highly expressed in microglia and monocytes (Figure 6F). Based on the UMAP and violin plots, microglia and monocytes had the highest senescence scores, as shown in Figure 6G.

### Intercellular communication and internal signaling based on scRNA-seq

**Figure 7:**
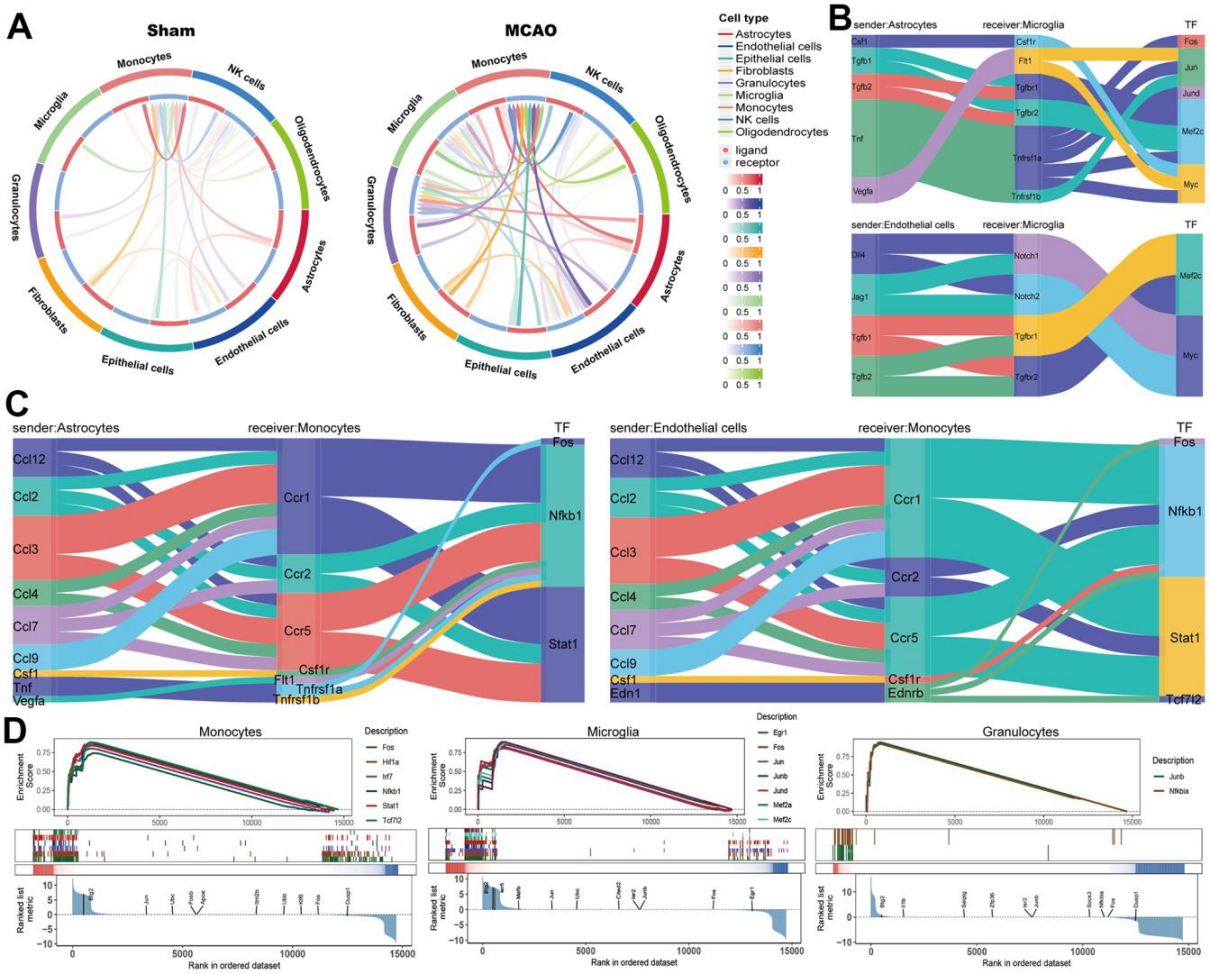
**Intercellular communication analysis based on scRNA-seq.** (**A**) The intercellular communication in the MCAO and sham groups. Microglia (**B**) and monocytes (**C**) as receivers assessed the cellular interactions with astrocytes and monocytes. (**D**) The TF enrichment plot in monocytes, microglia, and granulocytes.

The intercellular communication in the MCAO and sham groups is shown, respectively (Figure 7A). Monocytes, granulocytes, and microglia were more involved in cellular communication as receptors in the sham group. Subsequently, we identified microglia and monocytes as receivers and assessed their cellular interactions with astrocytes and monocytes (Figure 7B, 7C). Further analysis of transcription factors (TFs) involved in cellular communication revealed that *Mef2c* and *Myc* were activated when microglia served as recipients, whereas *Fos*, *Nfkb1*, and *Stat1* were activated when monocytes served as recipients. Finally, we presented TF activities in receiver cells using a TF enrichment plot, and all TFs were activated in monocytes, microglia, and granulocytes (Figure 7D).

### Cell trajectory analysis of microglia

**Figure 8:**
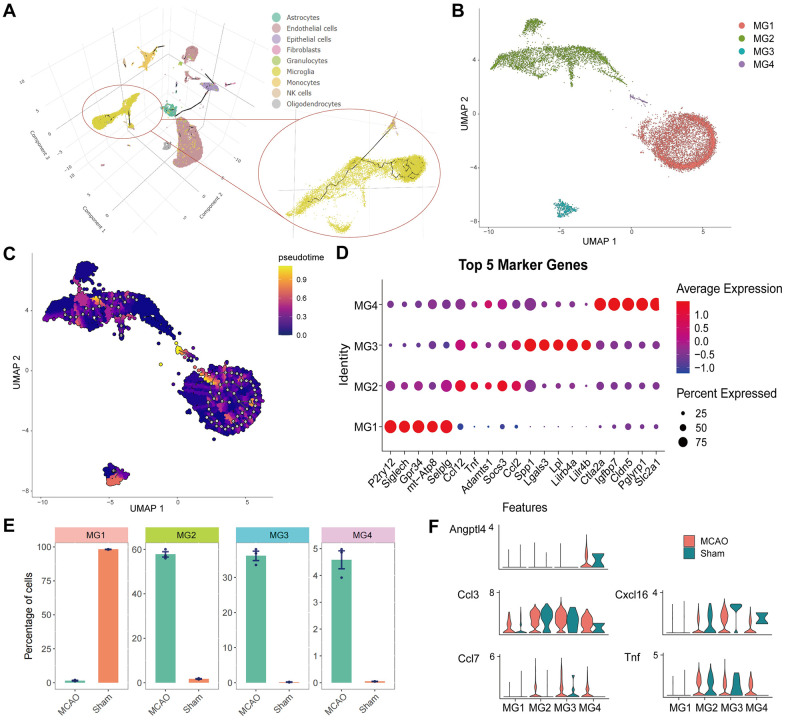
**Cell trajectory analysis and identification of microglia.** (**A**) 3D images of cell trajectories and the microglia part are amplified. (**B**) Individual clustering and UMAP mapping for microglia. (**C**) Cell trajectories of the microglia. (**D**) The bubble pattern of the top five cell marker genes in four microglia clusters. (**E**) Distribution of four cell clusters in the IS and Sham groups. (**F**) Quantified expression of HSRGs in four cell clusters.

The cell trajectories of the microglia are presented as 3D images in Figure 8A. Individual clustering and UMAP mapping showed that microglia were divided into four clusters (Figure 8B). To annotate these microglial cell sub-clusters, we identified the top five cell marker genes in each cluster (Figure 8D). The marker genes include *“P2RY12”, “SIGLECH”, “GPR34”,* “*mt-ATP8*”, “*SELPLG*” of MG1, *“CCL12”, “TNF”, “ADAMTS1”, “SOCS3”, “CCL2”* of MG2, *“SPP1”, “LGALS3”, “LPL”, “LILRB4A”, “LILR4B”* of MG3, and *“CTLA2A”, “IGFBP7”, “CLDN5”, “PGLYRP1”, “SLC2A1”* of MG4. Analysis of the differences in the number of cell clusters showed that MG1 was the main microglia in the sham group, whereas MG2, MG3, and MG4 were the main microglia in the MCAO group (Figure 8E). Additionally, *ANGPTL4* showed specificity for MG4, and its expression was higher in the MCAO group. In MG2-4, *CCL7*, *CXCL16,* and *TNF* were highly expressed in the MCAO group. Although *CCL3* was expressed in different subgroups, its expression level was higher in the MCAO group (Figure 8F). Finally, the cell trajectory of microglia was determined to explore their divergent trajectory.

### Drug screening and molecular docking

**Figure 9:**
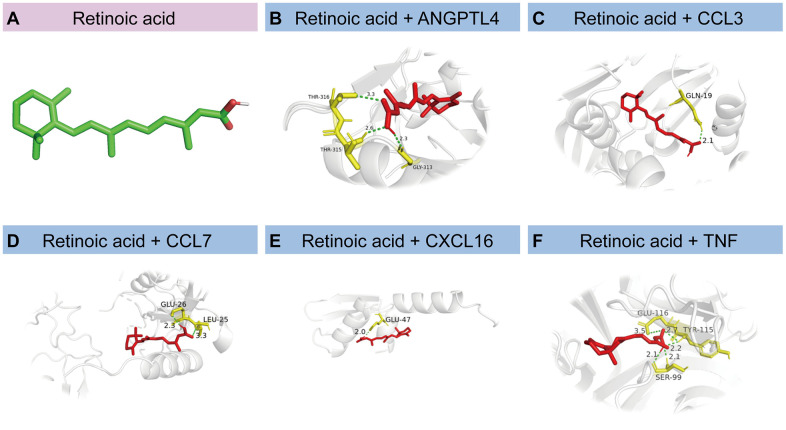
**Molecular docking of proteins corresponding to HSRGs and retinoic acid.** (**A**) The structural diagram of retinoic acid. (**B**–**F**) Docking simulation of proteins and small molecule compounds.

Small-molecule compounds that may bind to *ANGPTL4*, *CCL3*, *CCL7*, *CXCL16,* and *TNF* were identified using the DSigDB database; the top 10 compounds are listed in Table 3. Among these, retinoic acid had the highest combined score (608812). We then drew a structural diagram of retinoic acid, which can bind to *ANGPTL4*, *CCL3*, *CCL7*, *CXCL16,* and *TNF* (Figure 9A–9F).

**Table 3:** The top 10 compounds bind to HSRGs.

**Term**	**Adjusted P-value**	**Combined score**	**Genes**
Retinoic acid	0.012148	608812	*CCL7, CCL3, ANGPTL4, TNF, CXCL16*
Roflumilast	0.001246	15085.69	*CCL3, TNF*
indinavir	0.001246	13656.83	*CCL3, TNF*
PCI-24781	0.001246	12459.14	*CCL7, CCL3*
Lopinavir	0.001609	8557.58	*CCL3, TNF*
Antimycin A	0.002311	5832.819	*CCL3, TNF*
isoproterenol	5.20E-04	5550.691	*CCL7, CCL3, TNF*
Honokiol	0.002577	5105.231	*CCL3, TNF*
15-Acetyldeoxynivalenol	5.20E-04	4787.728	*CCL3, ANGPTL4, TNF*
palmitic acid	0.003584	3921.078	*CCL3, TNF*

## DISCUSSION

Senescence has long been a significant issue for researchers and has accelerated since the occurrence of IS [6]. Cellular senescence is one of the significant causes of senescence and has recently attracted considerable attention [11]. SenMayo is a set of genes that accurately describes and assesses cellular senescence [16]. In this study, we aimed to identify the HSRGs involved in IS and cellular senescence in brain tissue by the SenMayo gene set. A nomogram model was constructed based on HSRGs and was evaluated preliminarily to predict cellular senescence in patients with IS. Immune activity plays a vital role in cellular senescence after IS, which has been discussed in detail using immune cell infiltration analysis. To further elucidate the mechanism, scRNA-seq analysis was performed to determine the cellular localization of HSRGs, intercellular communication, and cellular trajectory. Finally, small molecules that can bind to hub gene expression proteins are considered potential drugs for alleviating cellular senescence after IS. Overall, this study combined multiple bioinformatic analysis methods and experimental verification to conduct a rigorous discussion of cellular senescence after IS at different transcriptome levels, providing a reference for further research in this field.

Functional enrichment analysis revealed that SRGs were primarily related to leukocyte migration and cytokine-cytokine receptor interactions. This suggests that the immune response plays a significant role in cellular senescence after an IS. However, while the immune response can be protective, the invasion of innate immune cells into the brain and meninges during the acute phase can exacerbate ischemic damage [25]. Additionally, peripheral organs can become a second “battlefield” for the immune response after IS. Danger signals are released from damaged brain cells into the circulatory system, which then activates systemic immunity, causing severe immunosuppression, life-threatening infections, and a poor prognosis [26]. In the chronic phase, antigen presentation initiates an adaptive immune response against the brain, which may underlie the neuropsychiatric sequelae [25]. Studies have also shown that microglia, astrocytes, foam cells, and lymphocytes are activated in IS, forming glial scars that persist for ten years later and are associated with cognitive decline [27]. During the acute phase of IS, a significant number of injured immune cells secrete various cytokines, while some cells exhibit a SASP pattern, which is an important indicator of cellular senescence after IS [28].

After validating with rat and human blood samples and RT-qPCR of rat brain tissue, *ANGPTL4*, *CCL3*, *CCL7*, *CXCL16*, and *TNF* were identified as HSRGs. *ANGPTL4* is a protein associated with endothelial cell integrity, inflammation, oxidative stress, and lipid metabolism and may be involved in the pathogenesis of IS [29]. *CCL3* and *CCL7*, as chemokines, and *CXCL16*, as chemokine ligands, are associated with the recruitment and activation of inflammatory cells, neuronal survival, and neoangiogenesis, and are important mediators of IS [30]. Previous studies have reported that *CCL3* may play an important role in neutrophil recruitment and the development of atherosclerosis [31]. Moreover, Waśkiel-Burnat et al. recently reported that *CCL7* may be a significant biomarker of atherosclerosis [32]. Additionally, *CXCL16* is implicated in the immune inflammatory response to atherosclerotic plaques, from antigen recognition to the migration and infiltration of immune cells into areas of inflammation [33, 34]. At the same time, *TNF* is not only associated with neuroinflammation after IS but also promotes SASP-stimulated lysosomal extravasation, leading to cellular senescence [35]. Sequencing data from the brain tissue in the MCAO model demonstrated good agreement with our PCR validation results, but a degree of variability was observed in the peripheral blood. For the lack of data from human brain tissue, we combined data from human peripheral blood to determine HSRGs. In human peripheral blood, *CCL3* and *TNF* showed decreased expression after IS, which may be related to the blood-brain barrier. However, it still indicates that the expression changes of HSRGs after IS are more sensitive than other SRGs. Moreover, we noticed that the expression of *Cxcl16* and *Tnf* in rat peripheral blood was different from that in humans, and the Temporal analysis showed that their expression trend changed again 6 hours after MCAO. We supposed that the difference is due to different stages of disease development at different points in time. Overall, the HSRGs we identified were species-conserved and showed some efficacy in predicting the onset of IS.

Our study also suggests that HSRGs expression may change over time and that predictive models may need to be adjusted over time. After IS, damaged neuronal cells release large amounts of senescence-associated cytokines that affect immune cell function [13]. Therefore, we focused on the different immune cell type expressions in the peripheral blood of patients with IS. Notably, the IS group had lower numbers of CD8+ T cells and activated NK cells. Additionally, HSRGs were significantly differentially expressed in neutrophils, naïve B cells, CD8+ T cells, and T-cell follicular helper cells, particularly in neutrophils and CD8+ T cells, where all hub genes were differentially expressed. CD8+ T cells in the peripheral blood migrate to the brain parenchyma after IS [36]. Ritzel et al. recently reported that CD8+ T cells enhance inflammation and leukocyte recruitment and act as a marker of senescence of the central nervous system [37]. Moreover, the majority of research suggested that the dynamics of NK cells in IS are characterized by an increase in the brain and a decrease in the peripheral blood, which was consistent with our results [38, 39]. Meanwhile, brain ischemia weakens NK cell-mediated immune defenses by interfering with neurogenic and intracellular pathways [40].

Among the various cell types in IS, microglia and monocytes are prone to show a cellular senescence phenotype in the brain tissue (Figure 6). Microglia play a key role in IS as resident central nervous system immune cells and are a double-edged sword for neural healing [41, 42]. Raffaele et al. have shown that microglia release microcytes that enhance the prognosis of IS by limiting the senescence of immune cells and promoting the formation of oligodendrocytes [43]. Furthermore, senescence-associated microglia can substantially affect brain homeostasis, particularly iron storage and metabolism, leading to senescence-related susceptibility and poor functional recovery after IS [44, 45]. Several studies have also indicated that the cellular senescence of monocytes is an important feature of immune-senescence that can delay or accelerate the establishment of atherosclerotic plaques [46, 47]. The present study further resolved the issue of communication between these cells and glial cells. We found that the intensity of cellular communication between granulocytes, microglia, and monocytes, which act as receivers in the MCAO group, was significantly increased compared to other cells, further illustrating the important role of the immune response after IS (Figure 7A). Taken together, we suggest that intervention in the cellular senescence phenotype of immune cells, especially microglia and monocytes, may be the key to reducing senescence and improving the prognosis of IS.

Microglia are intrinsic brain cells that are important for senescence. After subpopulation analysis of microglia, MG4 was found to be closely related to cellular senescence owing to higher levels of hub gene expression in the MCAO group. Based on marker genes, MG4 cells were identified as vessel-associated microglia, maintaining blood-brain barrier integrity via Claudin-5 expression, a tight-junction protein. Vessel-associated microglia maintain BBB integrity at first by expressing the tight-junction protein Claudin-5 and making physical contact with endothelial cells, while microglia phagocytose astrocytic end-feet and disrupt BBB function during chronic inflammation [48]. Furthermore, retinoic acid was identified as a small compound that could bind to HSRGs. The positive effect of retinoic acid in improving the prognosis of IS possibly relies on improving blood-brain barrier disruption and reducing apoptosis and neuronal damage, which has been demonstrated in animal studies but is still lacking in clinical studies [49–51]. The reversal effects of retinoic acid on cellular senescence phenotypes have also been documented [52, 53]. We believe that retinoic acid could be used as a possible medication to ameliorate the cellular senescence phenotype and improve the prognosis of IS.

Even though we rigorously discussed the senescence of various cell types after IS, this study has some limitations. First, although a clinical prediction model constructed based on HSRGs was verified, further verification using external data is lacking. Additionally, vessel-associated microglia have been identified to play an important role in cellular senescence, and further flow cytometry to verify their function. Moreover, owing to the lack of data from neuronal cells, the cellular senescence of neurons after IS was not discussed in this study. Finally, retinoic acid has been identified as a potential drug for improving the cellular senescence phenotype and prognosis of IS; however, further experimental validation is required.
